# A commentary on ‘Causal effects of gut microbiota on renal tumor: a Mendelian randomization study’

**DOI:** 10.1097/JS9.0000000000001704

**Published:** 2024-05-29

**Authors:** Xingcheng Zhu, Qiuyu Mao, Junxian Zhao, Peiqin Zhan, Zhengyan Wang, Tianci Gao, Hongping Bao

**Affiliations:** a Department of Clinical Laboratory, The Second People’s Hospital of Qujing City, Qujing; b Department of Urology, The Second Affiliated Hospital of Kunming Medical University, Yunnan; c Department of Urology, 920th Hospital of Joint Logistics Support Force of Chinese People’s Liberation Army, Kunming, Yunnan Province; d The Second Hospital of Jilin University, Jilin; e Department of Gastrointestinal Surgery, The Second People’s Hospital of Qujing City, Qujing, Yunnan Province, People’s Republic of China

Highlights

- Large-scale two-sample Mendelian randomization (MR) analyses necessitate false discovery rate (FDR) correction.
- The latest database update for gut microbiota encompasses 473 species.
- In addition to conventional heterogeneity and pleiotropy tests [Mendelian Randomization Egger (MR-EGGER) and Mendelian Randomization Pleiotropy RESidual Sum and Outlier (MR-PRESSO)], Bayesian-weighted Mendelian Randomization should also be considered.
- After Venn diagram analysis, a transcriptomic assessment should be performed to find comorbid genes.

*Dear Editor,*

We have thoroughly reviewed the article by Zhang *et al*.^1^ published in the *International Journal of Surgery*. They employed traditional two-sample Mendelian Randomization (MR) to elucidate certain relationships between gut microbiota and renal tumors, identifying specific gut bacterial species that either protect against or increase the risk of benign and malignant renal tumors. We commend the authors for their exemplary work and innovative approach. However, we have several observations and suggestions to share concerning this topic.

Firstly, in conducting large-scale two-sample MR analyses, it is crucial to implement false discovery rate (FDR) corrections. Given that each analysis is accurate at a 95% confidence interval, the probability of achieving correct *P* values across multiple analyses diminishes significantly. For instance, after the third MR analysis, the probability of all three being correct is 85.7% (95%^3). Therefore, FDR correction is essential following extensive MR analyses.

Secondly, the database used by the authors is somewhat outdated. As of 2024, the database for gut microbiota has been expanded from 211 to 473 species. To ensure a more comprehensive analysis of causal relationships, it is advisable to utilize this updated database.

Thirdly, while the authors employed various tools to assess heterogeneity and pleiotropy, the inclusion of Bayesian-weighted MR should be considered with large-scale sample sizes. In large-scale MR analyses, there may be significant variations in the explanatory power of genetic instrumental variables and their association strength with the outcome variables. Bayesian-weighted MR, by appropriately weighting each instrumental variable (based on statistical significance and effect size), can enhance the accuracy of the overall model estimation.

Lastly, the authors identified certain gut microbiota species associated with both benign and malignant tumors through Venn diagrams. We believe this finding is meaningful and further suggest that the corresponding single nucleotide polymorphisms of these shared gut microbiota species be converted into relevant genes via https://biit.cs.ut.ee/gprofiler, followed by standard transcriptomic analyses to identify key genes that may contribute to comorbid conditions.

In summary, we are grateful for the efforts of Zhang *et al*. in exploring the causal relationship between gut microbiota and renal cancer. However, we believe that our suggestions could refine the accuracy of their results and provide deeper insights for meaningful experimental outcomes.

## Ethical approval

Not applicable.

## Consent

Not applicable.

## Source of funding

Not applicable.

## Author contribution

X.Z., Q.M., and J.Z.: design, literature search, and writing; P.Z.: literature search and data collections; Z.W., T.G., and H.B.: providing language polish.

## Conflicts of interest disclosure

The authors declare that they have no conflicts of interest.

## Research registration unique identifying number (UIN)

Not applicable.

## Guarantor

Xingcheng Zhu.

## Provenance and peer review

Not applicable.

## Data availability statement

All data generated and analyzed during this study are included in this published article. The data presented in the article may be requested by consulting the correspondence author.

## References

[1] Zhang F Xiong Y Zhang B . Causal effects of gut microbiota on renal tumor: a mendelian randomization study. Int J Surg 2024;110:1870–1872. 38224351 10.1097/JS9.0000000000001041 PMC10942221

